# Facilitating patient-centred care for special care dentistry patients: A Quality Improvement Project in the Community Dental Service

**DOI:** 10.1038/s41405-020-0038-4

**Published:** 2020-07-29

**Authors:** Aoife Nic Iomhair, Miriam John

**Affiliations:** 1 Dental Core Trainee, School of Dentistry, Belfast Health and Social Care Trust, Belfast, UK; 2 LINCymru Clinical Lead, Quality Improvement Skills Training Section, Health Education and Improvement Wales (HEIW), Cardiff, UK

**Keywords:** Special care dentistry, Dental public health

## Abstract

**Aim:**

A Quality Improvement Project in one of the Special Care Dentistry (SCD) specialist centres in the School of Dentistry in Belfast aimed to improve the identification of the specific dental care needs of SCD patients from information available in the clinical record.

**Methods:**

The Model for Improvement was used to define project objectives and subsequently implement changes to practice aimed at achieving those objectives through Plan-Do-Study-Act (PDSA) cycles. Continuous data collection to monitor performance was undertaken throughout.

**Design:**

A Patient Needs Assessment Tool to be used during new SCD patient assessments was designed and introduced, with subsequent changes to the defined process for use following analysis of the success of the initial intervention.

**Results:**

The amount of information on relevant dental care needs identifiable from clinical records following new SCD patient assessments increased from 30 to 90%, whereas the time taken to identify the relevant information decreased from 2 min and 31 s to 17 s.

**Conclusion:**

The use of a Patient Needs Assessment pro-forma can achieve significant improvements in the extent and accessibility of information available to assist in the planning and delivery of appropriate and equitable care for SCD patients.

## Problem

Special Care Dentistry (SCD) is defined as the branch of dentistry which ‘provides preventive and treatment oral care services for adults who are unable to accept routine dental care because of some physical, intellectual, medical, emotional, sensory, mental or social impairment, or a combination of these factors.’ It is the most recently established of the 13 dental specialities, having been officially recognised by the General Dental Council in September 2008.^1^

Patients referred to SCD services require individualised adaptations to the conventional provision of dentistry, being unable to accept routine dental care due to any or a combination of the potential factors listed above. The provision of equitable dental care for this group of patients can often require any number and means of modifications to conventional treatment planning, dependent on the individual patient and their specific needs.

Attaining consistent high-quality care for patients with additional needs, at a standard equivalent to that afforded to the general population, first requires identification and clear documentation of each patient’s specific disability, impairment or limitation, the implications that this could have on our provision of dental care and the modifications that are required to ensure the achievement of equitable care.^2^

A comprehensive, holistic assessment and thorough documentation of all potential factors that may impact on their ability to receive care and our ability to deliver care should allow planning and preparation for safe, appropriate and equitable care. The ready availability of such information to all clinicians involved would assist in the provision of care which meets all six of the criteria defined by the National Academy of Medicine, formerly the Institute of Medicine, as markers of highquality care; those being safe, effective, equitable, efficient, timely and patient-centred.^3^

Discussions among colleagues at one of the SCD specialist centres within the North Wales Community Dental Service (NWCDS) revealed difficulties in identifying relevant information on patient-specific dental care needs from the clinical records of SCD patients in a timely and efficient manner.

It was suggested that this could present challenges in attempting to meet the individualised dental care needs of these patients, especially for new and inexperienced members of staff, due to an inability to implement appropriate planning and preparation for appointments. This was identified as a potential area for improvement.

## Background

### Setting

Betsi Cadwaladr University Local Health Board (BCUHB), making up almost one third of the landmass of Wales and serving a population of ~694,000 is the largest local health board in Wales, in terms of both population and geography. It covers the six main areas in North Wales; Anglesey, Conwy, Denbighshire, Flintshire, Gwynedd and Wrexham providing a full range of primary, community and secondary care services.^4^

Within BCUHB, the North Wales Community Dental Service employs over 200 staff across 27 clinics, health centres and community hospitals, with ten dentists on one or more of the GDC Specialist lists.

The role of the Community Dental Service (CDS) is to provide specialised/specialist services on referral and routine dental care for vulnerable patients of all ages who are unable to access alternative dental services. The patient groups catered for include patients with physical and learning disabilities, medically compromised patients, patients with mental health needs, anxious patients, housebound patients, patients in geographical isolation, patients in rehabilitation and secure units and those with complex social issues.^5^

Figures relating to CDS activity 2018–2019 from the Welsh Government indicate that 39% of patients treated within the NWCDS in BCUHB during that period met the criteria for the provision of SCD services.^6^

The Quality Improvement Project described was initiated at Holywell Community Dental Clinic in Flintshire, with the aim of expanding the intervention throughout the whole service following successful planning and implementation on a small scale.

### Special Care Dentistry

The World Health Organisation’s International Classification of Functioning, Disability and Health (ICF), describes people who require Special Care Dentistry services as those with a ‘disability or activity restriction that directly or indirectly affects their oral health, within the personal and environmental context of the individual.’^7^

Research has suggested that people with disabilities and those living with long-term disabling conditions are affected by poorer levels of oral health than their non-disabled counterparts. It has been found that although disease experience is broadly similar in disabled and non-disabled individuals, people affected by disabilities are less likely to have oral disease treated and when treated, are more likely to receive extractions than restorative treatment. The evidence would suggest that the poorer oral health outcomes experienced by disabled people are more likely to be as a result of barriers to accessing dental services and receiving dental treatment.^8–11^

An extensive range of potential barriers which may prevent disabled people from accessing dental care and maintaining their oral health in an equitable manner to non-disabled people have been identified, including; difficulties with communication, social isolation, lack of suitable transport arrangements, poor mobility, sensory impairments, cognitive impairment, anxiety, poor manual dexterity, physical inability to tolerate conventional dental treatment and learning disability.^12^

The Equality Act UK 2010 requires all service providers, including dental professionals, not to discriminate against people with disabilities by providing inadequate facilities or services at a poorer standard or in a worse manner than would be available for other members of the population.^13^

As a service providing care for a large number of SCD patients with additional needs, the NWCDS recognises the need to conduct a thorough assessment of potential barriers that may prevent each SCD patient under their care from achieving optimum oral health. This is an essential first step in attempting to minimise or remove these barriers. It was from this concept that the idea for this Quality Improvement Project was originally developed.

### Patient-centred care

Within the field of healthcare, quality has been defined as; ‘the degree to which health services for individuals and populations increase the likelihood of desired health outcomes and are consistent with current professional knowledge’.^3^

As referenced, the National Academy of Medicine have outlined six dimensions of healthcare quality, including the idea of ‘patient-centred care’.^3^ The concept of ‘patient-centred care’ is a complex and evolving area, which involves collaboration between patients and healthcare providers to ensure that the delivery of care takes account of and respects the specific needs and preferences of the individuals concerned.^3^

Rather than attempting to provide a limited definition of the concept, The Health Foundation have outlined four principles of ‘patient-centred care’:^14^

1. Affording people dignity, compassion and respect
2. Offering coordinated care, support or treatment
3. Offering personalised care, support or treatment
4. Supporting people to recognise and develop their own strengths and abilities to enable them to live an independent and fulfilling life

The concept of ‘patient-centred care’ is at the heart of SCD as a specialty.

### Improving Quality Together

This Quality Improvement Project was undertaken as part of Health Education and Improvement Wales’s (HEIW) Quality Improvement Skills Training (QIST) programme for Silver Level Improving Quality Together (IQT) Accreditation.

Improving Quality Together is the Welsh national quality improvement training programme for National Health Service (NHS) Wales staff, trainees, students and educators, consisting of three levels of training; bronze, silver and gold. The programme, which is delivered by quality improvement teams across each health board in Wales was developed by *Improvement Cymru* with the goal of providing training in a common and consistent quality improvement methodology.^15^

**Fig. 1:**
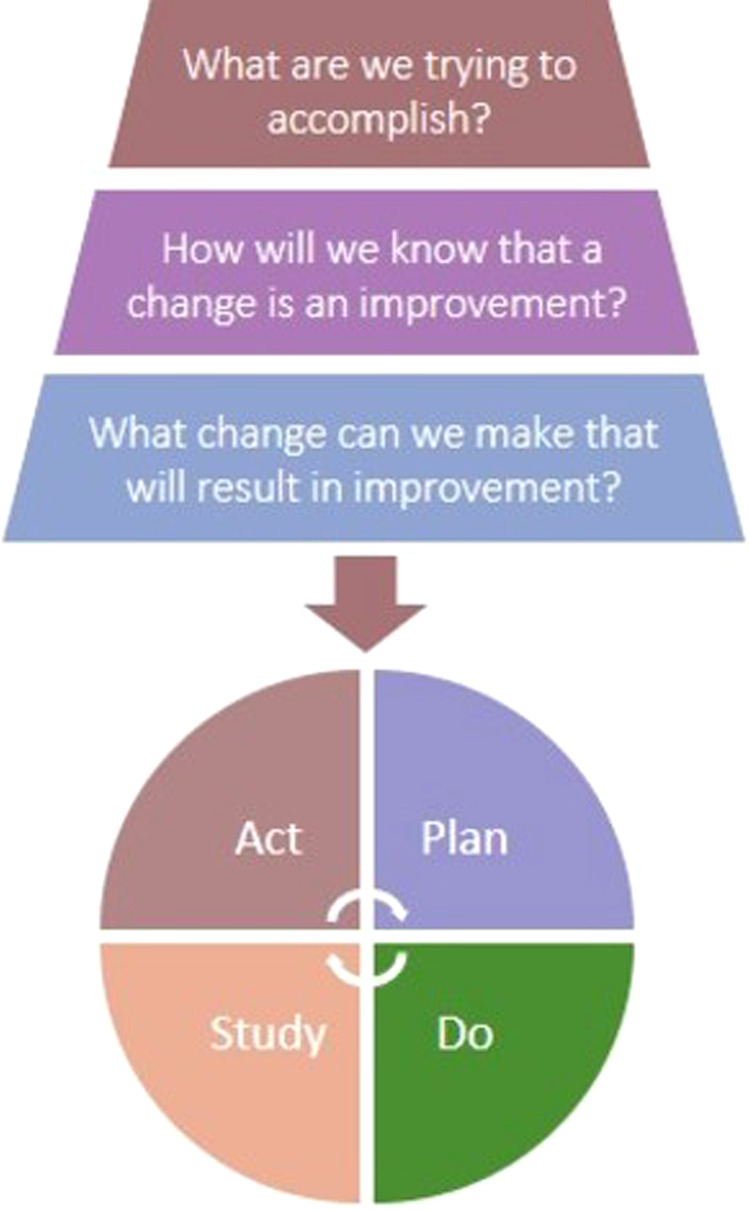
The Model for Improvement. The Model for Improvement is the quality improvement methodology used in the IQT programme. It aims to achieve improvements in healthcare systems, processes and outcomes using a standardised two-phase methodology; three questions asked and addressed in any order to define the nature of the proposed project, followed by the implementation of Plan-Do-Study-Act (PDSA) cycles to test the impact of changes on performance.

The Model for Improvement aims to achieve improvements in healthcare systems, processes and outcomes using a standardised two-phase methodology; three questions asked and addressed in any order to define the nature of the proposed project, followed by the implementation of Plan-Do-Study-Act (PDSA) cycles to test changes, while continually monitoring data to assess for improvements in performance (Fig. 1).^16^

## Aim

The overarching aim of this project was to try and enable the efficient and effective identification of the specific dental care needs of SCD patients in a CDS setting from information available in the clinical record.

It was hoped that this would allow the delivery of safe, appropriate and equitable care for these patients in a timely manner, regardless of potential barriers or additional needs and would ultimately reduce the potential for unmet dental care needs amongst our most vulnerable patients.

## Measurement

### Setting up measures

Having defined a broad improvement aim for the project, the next step involved setting up measures and establishing baseline performance against which to measure whether change resulted in improvement.

To enable measurement, a standardised criterion of the information which should be assessed for and included in the clinical record of a SCD patient was established, allowing performance to be measured against this standard.

Discussions and brainstorming sessions with specialists in the field of SCD and other clinicians experienced in the treatments of patients with additional needs were held to collate ideas on the factors which should be assessed and documented during an initial assessment of a SCD patient. A review of relevant literature on the subject was also carried out.^2,12,17,18^

The collaboration of findings from the literature and the views of experienced colleagues resulted in the identification of ten areas, information on which was suggested as being of importance to the effective dental care of SCD patients (Table 1). These findings advocated a holistic approach to the assessment of SCD patients, enabling the potential identification of barriers to maintaining oral health and receiving appropriate dental care within each patient’s life. Examples of such barriers included poor manual dexterity preventing effective oral hygiene behaviours, sensory impairments preventing successful communication with dental care professionals and social or geographical isolation inhibiting patients from being able to attend the dental clinic.

**Table 1:** Ten areas identified as of importance to the effective dental care of SCD patients.

Area requiring information	Examples
Next of kin	Family/carer contact details
Care group	Learning disability/physical disability
Medical history	Implications on oral health
Social circumstances	Supported living/carer support
Transport	Hospital transport required
Communication	Non-verbal/alternative communication methods required
Mobility	Walking aids/carer support required
Sensory impairments	Vision/hearing loss
Oral hygiene considerations	Reduced manual dexterity/carer support required
Dental treatment considerations	Treated upright/support cushions required/swallow issues

The most appropriate measures to assess baseline performance and subsequent changes were deemed to be; the amount of information on the specific dental care needs identified from the clinical record of each patient attending for a New Patient Assessment and the length of time taken to identify the necessary information from the record.

Individual clinical records were analysed for information relating to each of the ten areas identified. The number of the ten areas identified per clinical record was then calculated and converted to a percentage. The time taken to analyse the clinical record in attempting to identify the relevant information was also recorded.

### Data collection

To measure baseline performance, data were collected from the clinical records of all new SCD patients seen for an initial assessment appointment following referral into the service from 22/10/18 to 07/12/18.

The first change (PDSA 1) was introduced on 10/12/18, followed by a 12-week period of data collection to identify changes in performance in the areas detailed, during which a further change (PDSA 2) was introduced on 15/01/19.

Data collection was carried out by the project lead, also the author of this paper, through analysis of both paper and electronic clinical records relevant to the patient. Patients who had attended for New Patient Assessment appointments were identified using the appointment management computer software. Baseline data were collected retrospectively. Data were subsequently collected on a weekly basis for New Patient Assessments carried out during the preceding week.

The following data were collected per New Patient Assessment;

- Mean percentage of needs assessed per patient using developed criteria as standard.
- Time taken to analyse the clinical record in attempting to identify the relevant information.
- Percentage of clinical records containing information on each aspect of care provision identified as of importance.

### Baseline performance

Baseline performance measurements indicated that a mean of 30% of the specific dental care needs outlined in the developed criteria were identifiable from the clinical record following New Patient Assessments appointments, while the mean time taken to assess a clinical record to try and identify the relevant information was 2 min and 31 s. Information on Medical History and Care Group was consistently recorded during New Patient Assessments and easily located within the clinical record, while information on Transport and Communication was most difficult to identify on analysis of the record.

## Design

### Making the aim smart

Having assessed baseline performance, two more specific aims were then defined using the SMART criteria (specific, measurable, realistic, achievable, time-bound):

1. To increase the percentage of information on specific dental care needs identifiable from the clinical record of SCD patients following a New Patient Assessment from 30 to 70% in a 12-week period.

2. To reduce the time taken to analyse the clinical record to identify the relevant information from a mean of 2 min and 31 s to <1 min in a 12-week period.

#### Identifying the real issue

A variety of tools were implemented to analyse the problem in greater depth.

Root Cause Analysis (RCA) is a systematic process designed for use in ‘investigating and categorising the root causes of events with safety, health, environmental, quality, reliability and production impacts.’ It is designed to help identify not only what and how an event occurred, but also why it happened.^19^

A RCA was carried out, beginning with the overall issue of insufficient preparation to meet the individualised needs of SCD patients and the subsequent consequences of this for patients, carers and members of the dental team.

This identified the real issue as;

‘Lack of a universal protocol for assessing and documenting the specific dental care needs and necessary adaptations to care for SCD patients, to allow the sharing of information and appropriate planning of care.’

**Fig. 2:**
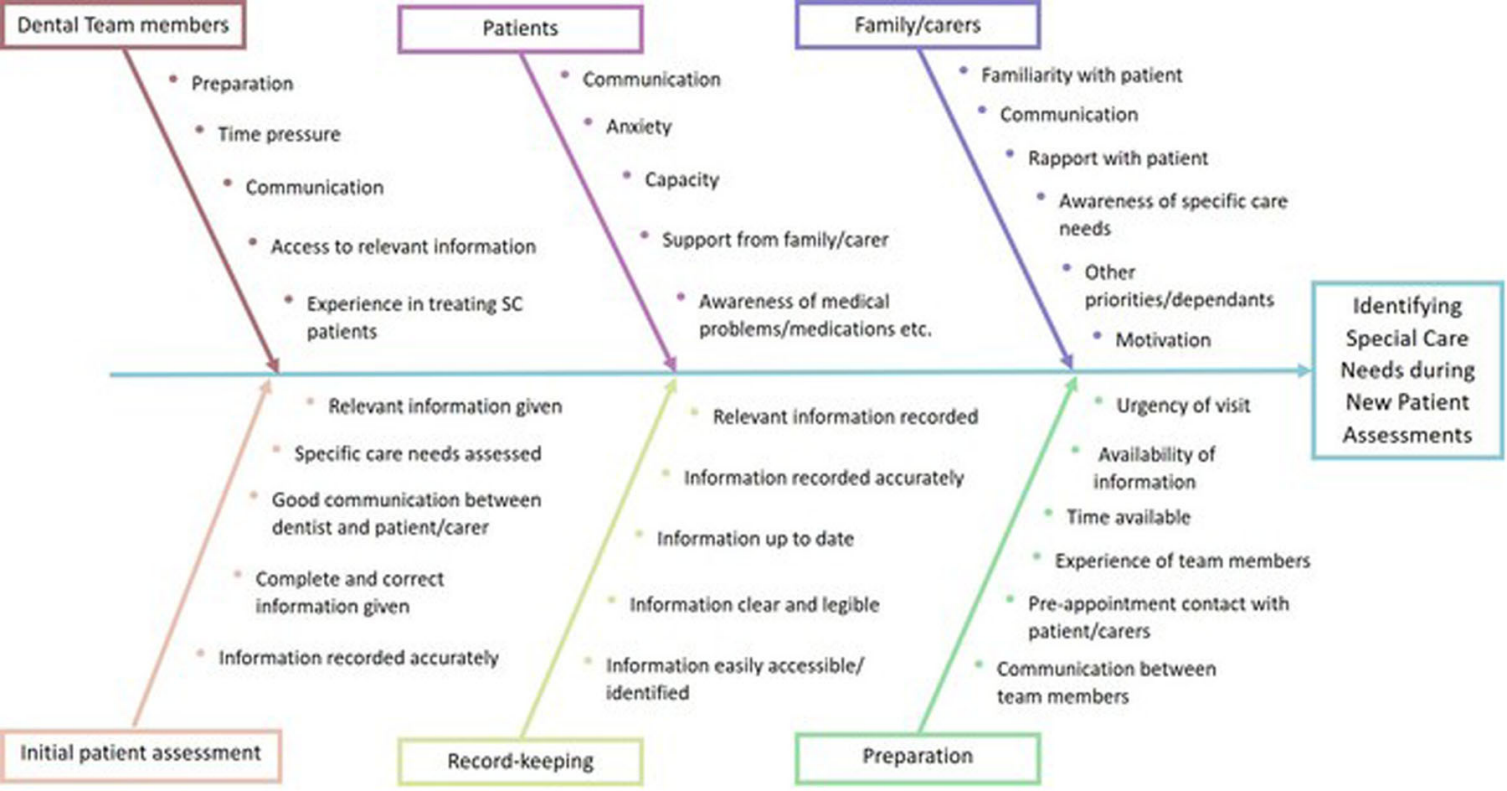
Ishikawa diagram. An Ishikawa diagram, also known as a ‘Cause and Effect’ diagram, aids quality improvement teams to explore and display potential causes contributing to an effect or outcome. This diagram shows the many potential factors within a variety of categories that could contribute to difficulties with identifying and preparing for the specific dental care needs of SCD patients.

**Fig. 3:**
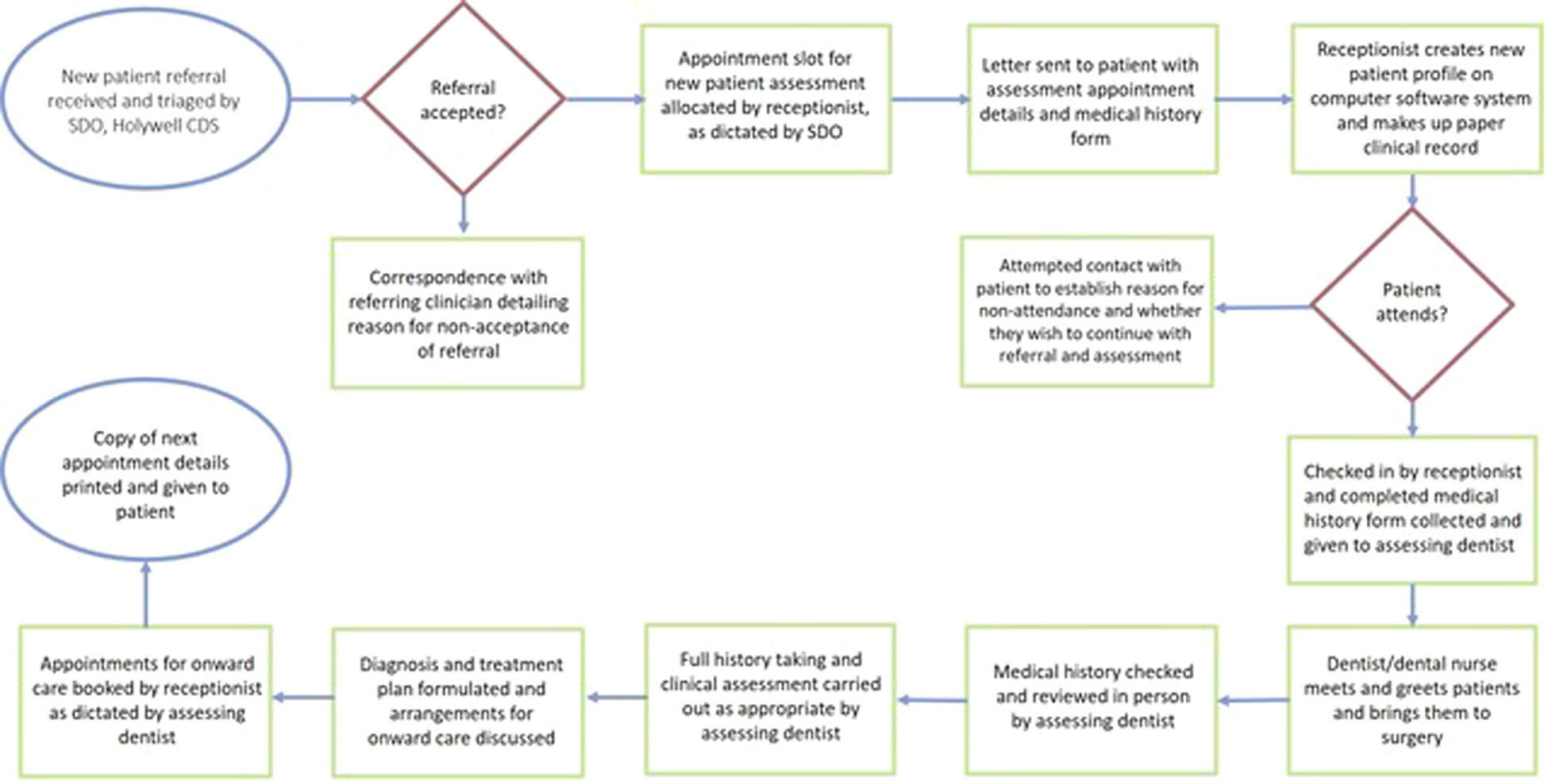
Process map. A process map is a visual representation of the sequence of steps in a process. Detailing the steps involved in a new patient referral into the SCD service helped to develop an understanding of how the process operated, in order to begin developing ideas about how to improve it.

An Ishikawa diagram^16^ was used to explore and display the many potential causes contributing to the difficulties in identifying and preparing for the specific dental care needs of SCD patients (Fig. 2). Additionally, a process map was devised detailing the steps currently involved in a new patient referral into the SCD service. This enabled ideas to be developed with regards to how the procedure could possibly be improved (Fig. 3).

### Options for change

**Fig. 4:**
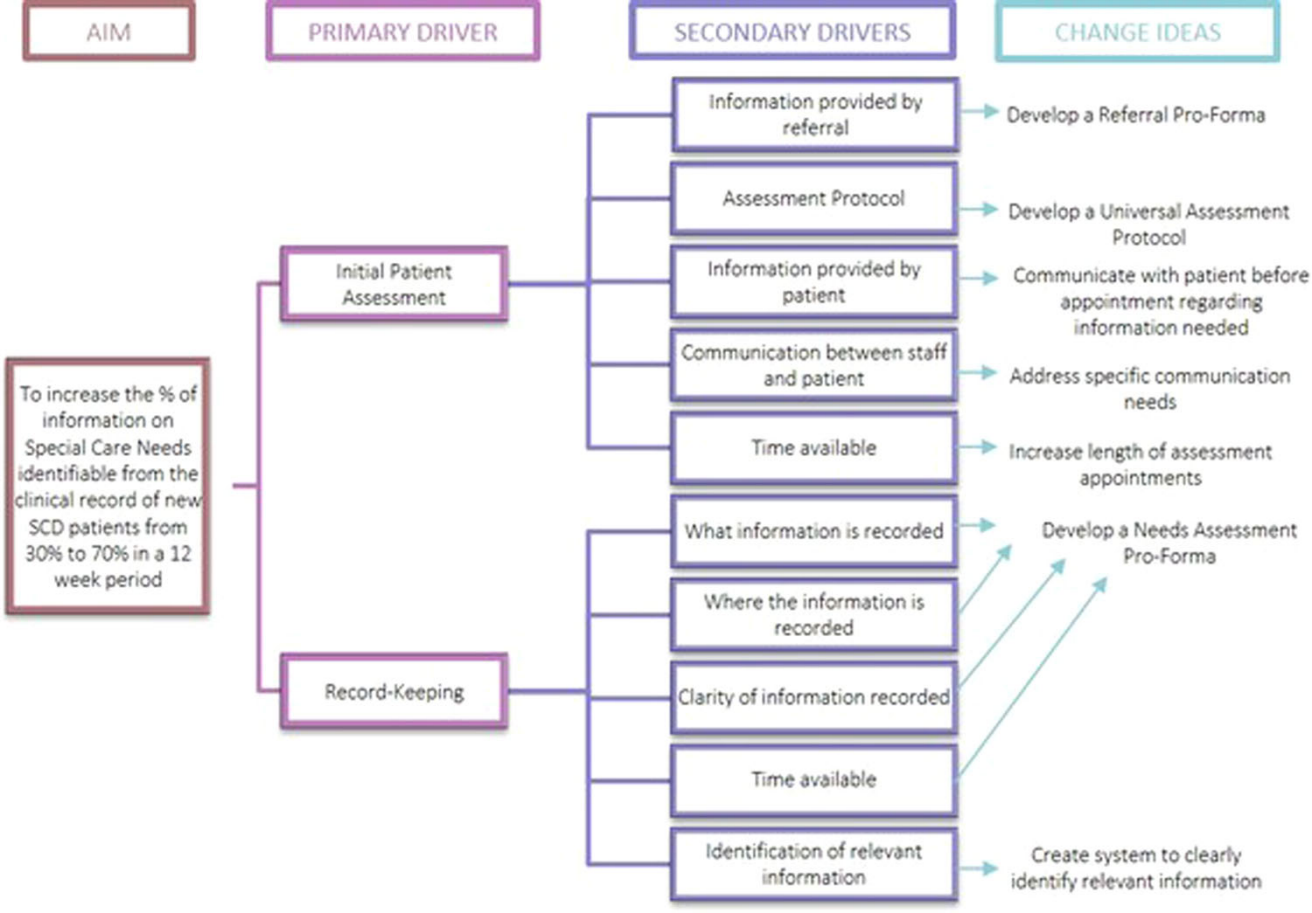
Driver diagram. A driver diagram was compiled to identify the primary drivers that would contribute directly to achieving the project aim, the secondary drivers which were components of the primary drivers, along with specific change ideas to test for each secondary driver.

A driver diagram was compiled in an attempt to identify potential changes that could lead to improvement (Fig. 4). Tools such as the Ease–Benefit Matrix and the Circle of Influence were used to identify the most appropriate option for initial change, which was deemed to be the introduction of a Patient Needs Assessment Tool to be used during new SCD patient assessment appointments.^16^

### Stakeholder involvement

The Stakeholder Analysis Tool from the NHS Quality Institute was used to identify stakeholders who could have a concern or interest in the project.^20^

The groups identified were SCD patients and their families and carers, CDS staff involved in SCD, SCD specialists and the North Wales CDS Directorate Group. Patients were involved through discussions regarding the purpose of the Patient Needs Assessment pro-forma and why the information required was important. Patients and carers provided the relevant information for completion of the Patient Needs Assessment pro-formas during the initial assessment appointments following referral into the service and their feedback on the project was encouraged.

North Wales CDS Directorate Group was approached for permission to carry out the project within the service and were updated on progress throughout. Members of the Directorate also contributed to the design, implementation and analysis of the project due to their specialist knowledge and experience, both in the field of Special Care Dentistry and in service delivery within a CDS setting.

Clinic staff were initially informed and subsequently updated on the project at Clinical Governance meetings, which involved all team members including receptionists, dental nurses, dental therapists, dental officers, a Dental Core Trainee and a specialist in SCD. Active contribution and feedback from all team members on the rationale for the project and the implementation of the proposed changes was encouraged.

### Ethical considerations

Given that the aim of the project was service improvement, the NHS Health Research Authority Decision Tool deemed that formal ethical approval was not necessary. To ensure patient confidentiality, data were collected using anonymous identifiers and data collection forms were stored securely on the BCUHB computer system.

## Strategy

### PDSA cycle 1

**Fig. 5:**
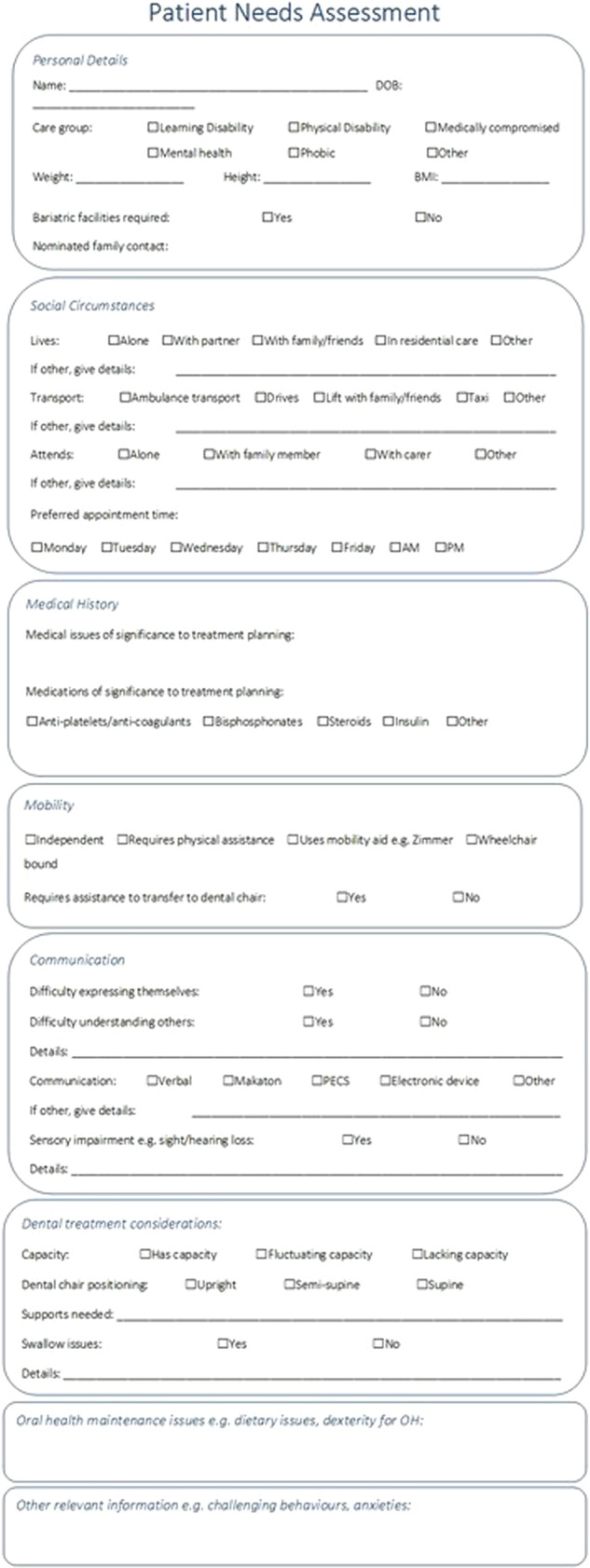
Patient needs assessment pro-forma. This pro-forma was designed to be completed during new SCD patient assessments to ensure that information on each of the ten areas identified as of importance to the care of SCD patients was collected during these initial assessments and was easily identifiable and readily located at susequent visits and by all members of the dental team.

PDSA 1 involved the design and introduction of the Patient Needs Assessment Tool in the form of a pro-forma. The design of the pro-forma was carried out by the project lead, in collaboration with specialists in Special Care Dentistry and members of the NWCDS Directorate. The pro-forma was designed to ensure that information on each of the ten areas of importance identified was collected at a New Patient Assessment appointment, was easily identifiable and readily located at subsequent visits and by all members of the dental team (Fig. 5).

This pro-forma was to be completed by the assessing dentist at each New Patient Assessment appointment during the process of history taking and information gathering. Blank copies of the pro-forma were available in each surgery within the clinic.

Following analysis of the data obtained during PDSA 1, a problem was identified with regards to assessing dentists remembering to complete the Patient Needs Assessment pro-forma during new SCD patient assessment appointments. This resulted in the pro-forma either not being completed or being completed retrospectively which resulted in incomplete collection of the information required.

### PDSA cycle 2

PDSA 2 tried to address the issue of reliance on memory to achieve completion of the Patient Needs Assessment pro-forma during new SCD patient assessment appointments.

The process was changed so that the pro-forma was printed on yellow paper and inserted into the clinical records of new SCD patients before the initial assessment appointments, thereby improving visibility and reducing reliance on memory. This meant that the assessing dentist was prompted to complete the pro-forma during the New Patient Assessment appointment when accessing the clinical record and increased the likelihood that the pro-forma would be completed contemporaneously.

## Results

### PDSA cycle 1

Monitoring performance following the introduction of the Patient Needs Assessment pro-forma showed that small improvements were achieved in the percentage of relevant care needs assessed for and documented during new SCD patient assessments.

There were also slight improvements in the time taken to assess each patient record in attempting to identify the relevant information and in the percentage of clinical records with each aspect of care provision assessed and documented.

However, performance was inconsistent and the target of 70% of relevant care needs assessed for and documented during new SCD patient assessment appointments was not achieved.

### PDSA cycle 2

Inserting a brightly coloured copy of the pro-forma into the patient’s clinical record prior to the New Patient Assessment appointment as a visual prompt for the assessing dentist resulted in significant improvements in the percentage of relevant care needs assessed for and documented, as well as the time taken to assess each patient record to identify the necessary information. The percentage of clinical records with each aspect of care provision assessed and documented also increased substantially.

**Fig. 6:**
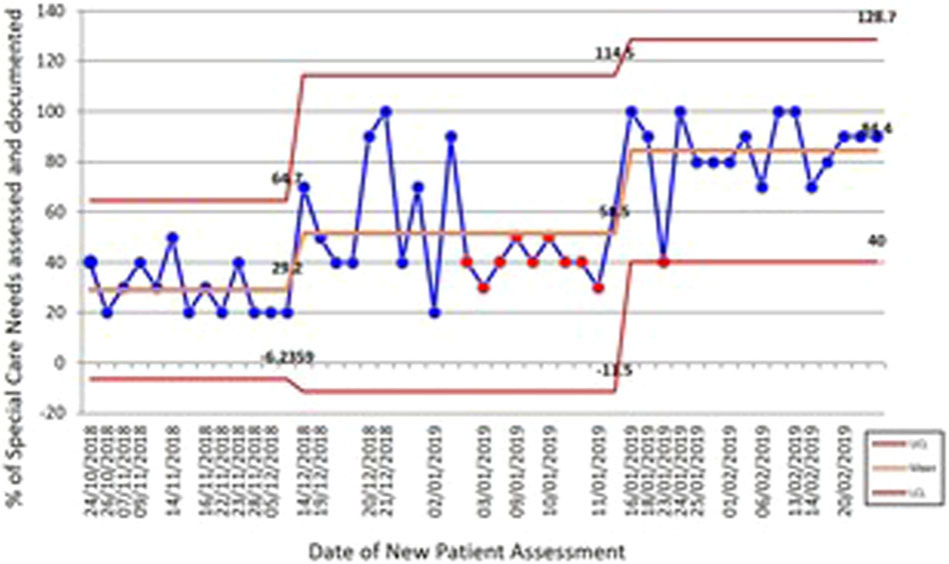
Statistical process control chart displaying data over time on the % of relevant care needs assessed for and documented during New Patient Assessments. Performance improved from a mean of  30% of relevant care needs assessed for new SCD patients at baseline to 90% after two cycles of change.

Performance improved from 30% of relevant care needs assessed for new patients at baseline to 90% after two cycles of change (Fig. 6).

The time taken to identify the relevant information decreased from a mean of 2 min and 31 s at baseline to a mean of 17 s following PDSA 2.

Information on each of the ten areas of care, apart from Family/Carer contact details was available from at least 70% of clinical records at the end of PDSA 2. Information on Family/Carer contact details increased from 8% of records at baseline to 31% following PDSA 2. This was the only area remaining below 70%.

## Lessons and limitations

This Quality Improvement Project has shown that the use of a Patient Needs Assessment pro-forma can achieve significant improvements in the extent and accessibility of information available to assist in planning and delivering appropriate and equitable care for Special Care Dentistry patients.

A pro-forma provides a written prompt as to the various aspects of care that should be recorded during a new SCD patient assessment and allows all the relevant information to be collated in one identifiable location, improving accessibility to the information required.

Human factors have a significant impact on the success of quality improvement initiatives.^21,22^ Reducing the potential for variability, reliance on memory and improving visibility has been shown in this project to allow improvements in the performance of individuals within a system.

Current barriers to the spread and sustainability of this quality improvement initiative include the additional time and administrative burdens presented by the completion of the Patient Needs Assessment pro-forma during an already busy initial assessment appointment. This may present an obstacle in attempting to implement this initiative on a CDS-wide basis in a sustainable way.

A potential resolution to this issue is the incorporation of the Patient Needs Assessment into the medical history form, some of which, e.g. next of kin details, transport requirements, social circumstances could be completed by the patient/carer prior to their appointment.

A multitude of quality improvement tools were utilised during the completion of this project, which enabled familiarisation with the variety of methodologies available. However, given the simplistic nature of the project, a more straightforward approach employing only the most relevant tools and methodologies could potentially have been adopted.

### Future recommendations

Further changes to streamline the process of collecting the necessary information and to reduce the additional time and administrative burdens described are required to enable the implementation of this initiative beyond the initial scope of the project.

A further PDSA cycle could include action on incorporating the assessment of specific dental care needs into the completion of the Medical History questionnaire, which is issued to patients/carers to be completed before the initial assessment appointment.

Involving patients in this way would also promote patient empowerment and engagement with their own care, key aspects of the overall concept of patient-centred care. For patients with impairments or disabilities precluding the completion of relevant aspects of the pro-forma, family members or carers could assist with this task. It would also be possible to utilise the many extended skills of all dental team members within the NWCDS, including dental nurses, many of whom have additional post-graduate qualifications in SCD, to assist patients with completion of the pro-forma prior to their appointment with the dentist.

The assessment of holistic factors that may impact on the provision, acceptance and risks of dental treatment is a particularly relevant issue in the SCD environment, given the additional needs of these patients, which warrant referral into such a service. However, it could be argued that this more holistic approach to patient care should be adopted for patients across all dental healthcare settings, to ensure that the care being provided is person-centred, tailored to individual needs and appropriate for the patient in question.

## Conclusions

This Quality Improvement Project has demonstrated an improvement in the amount and accessibility of information on the specific dental care needs of SCD patients available from clinical records through the implementation a of Patient Needs Assessment pro-forma. This was achieved using the Model for Improvement.

This project has shown that the creation of a consistent methodology for the assessment and documentation of patient-specific needs has the potential to achieve consistency in the information available to assist in the planning and provision of care.

The role of human factors has been highlighted as a key consideration in attempting to implement quality improvement initiatives. Strategies to minimise the effects of human factors on achieving improvements in performance may be required.

It is evident that the sustainability of a quality improvement initiative is critical to its success. As such, factors such as the efficiency and feasibility of suggested changes in a practical setting must be taken into account.

Finally, although the project described has achieved improvements which could theoretically assist with the delivery of patient-centred care, further work would be required to examine the actual impact of these improvements on the quality of care received by patients.
